# Aerosolization of Nanotherapeutics as a Newly Emerging Treatment Regimen for Peritoneal Carcinomatosis

**DOI:** 10.3390/cancers11070906

**Published:** 2019-06-28

**Authors:** Molood Shariati, Wouter Willaert, Wim Ceelen, Stefaan C. De Smedt, Katrien Remaut

**Affiliations:** 1 Laboratory of General Biochemistry and Physical Pharmacy, Faculty of Pharmaceutical Sciences, Ghent University, 9000 Ghent, Belgium; 2 Cancer Research Institute Ghent (CRIG), 9000 Ghent, Belgium; 3 Laboratory of Experimental Surgery, Department of Surgery, Ghent University Hospital, 9000 Ghent, Belgium

**Keywords:** peritoneal carcinomatosis, PIPAC, nebulization, nanomedicine, localized chemotherapy, intraperitoneal administration

## Abstract

Recent advances in locoregional chemotherapy have opened the door to new approaches for the clinical management of peritoneal carcinomatosis (PC) by facilitating the delivery of anti-neoplastic agents directly to the tumor site, while mitigating adverse effects typically associated with systemic administration. In particular, an innovative intra-abdominal chemotherapeutic approach, known as Pressurized Intraperitoneal Aerosol Chemotherapy (PIPAC), was recently introduced to the intraperitoneal (IP) therapy regimens as a palliative therapeutic option in patients with PC, presumably providing a better drug distribution pattern together with deeper drug penetration into tumor nodules within the peritoneal space. Furthermore, the progress of nanotechnology in the past few decades has prompted the application of different nanomaterials in IP cancer therapy, offering new possibilities in this field ranging from an extended retention time to sustained drug release in the peritoneal cavity. This review highlights the progress, challenges, and opportunities in utilizing cancer nanotherapeutics for locoregional drug delivery, with a special emphasis on the aerosolization approach for intraperitoneal therapies.

## 1. Introduction

Cancer is recognized as a major public health problem in the world today and is the second leading cause of death globally, exceeded only by cardiovascular diseases. Notwithstanding the fact that cancer mortality rates are declining annually in both men and women owing to improved treatment and organized screening, in 2019, 1,410,000 cancer deaths are projected to occur in the European Union (EU) alone [1].

Cancer involves the formation of tumors by abnormal cell growth that can occur in any organ of the body. The main cancer types are lung, prostate, colorectal, and stomach cancers for males, while females most often suffer from breast, colorectal, lung, and cervical cancer. Furthermore, cancer cells can spread and invade other tissues apart from the primary tumor site, leading to the formation of metastases in other parts of the body.

To date, cancer treatment still often relies on the implementation of conventional cancer chemotherapeutics that act by killing rapidly dividing cells. Chemotherapeutics like doxorubicin, taxanes (e.g., paclitaxel and docetaxel), or platinum-based agents (e.g., cisplatin, oxaliplatin, and carboplatin) are frequently administered by intravenous delivery. Then, chemotherapeutics depend on systemic transport to reach primary tumors or metastasis. The development of systemic chemotherapy regimens greatly improved the survival rate of patients, although considerable obstacles still exist that hinder the effective treatment of cancer. Systemic administration of chemotherapeutic agents in the bloodstream is generally accompanied by a low therapeutic efficacy and severe toxicity in healthy tissues ascribed to a short circulation half-life and off-target biodistribution, respectively [2]. In addition, only a fraction of the administered dose ends up at the tumor site and maintaining adequate drug levels within the tumors poses a significant challenge. Furthermore, the high compactness of most solid tumors impedes the deep penetration of chemotherapeutic drugs into the central regions of the tumor which, in turn, gives rise to only the partial eradication of cancer cells at the periphery of the tumor spheroid and around blood vessels [3,4]. Importantly, heterogeneities within tumors, as well as between patients referred to as intratumoral and intertumoral heterogeneity, respectively, present additional challenges to the development of effective personalized cancer therapies [5]. 

The locoregional administration of chemotherapeutics has great potential for addressing the drawbacks associated with systemic chemotherapy. Localized chemotherapy—defined as the application of chemotherapeutics directly to the target tissue—has several merits, such as the superior local drug concentration and reduced adverse effects on organs distant from the injection site [6,7]. Consequently, a reduced dosage can be applied for localized cancer therapy, together with the need for less frequent drug administration when compared to the intravenous administration route. 

Clearly, however, not all tumors are easily accessible for locoregional treatment and when they are, the first treatment option remains the surgical removal of the tumor nodules, whenever possible. One specific cancer type that might significantly benefit from locoregional therapy is peritoneal carcinomatosis (PC), originating from primary cancers of organs confined to the peritoneal cavity such as the ovary, prostate, and colon cancer. PC is characterized by the spread of numerous small tumor nodules (metastasis) onto the peritoneal membrane and is difficult to eradicate as the large surface of the peritoneal cavity (~2 m^2^) in conjunction with poor vascularization of the peritoneum restrain the potential of systemic chemotherapy for reaching the intraperitoneal (IP) tumor nodules. 

**Figure 1:**
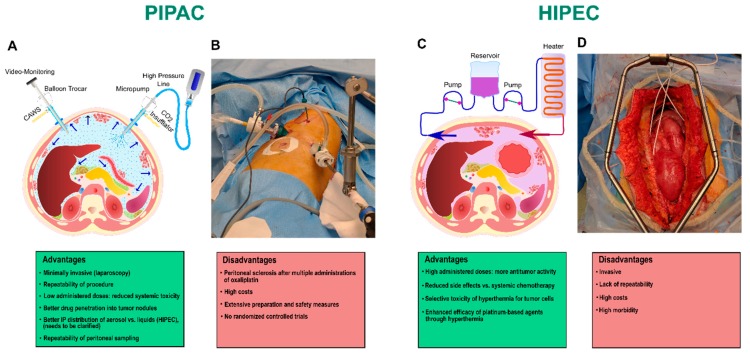
Examples of intraperitoneal chemotherapy methods along with representative advantages and disadvantages of each approach. (**A**) Schematic illustration and (**B**) surgical procedure of Pressurized Intraperitoneal Aerosol Chemotherapy (PIPAC). (**C**) Schematic illustration and (**D**) surgical procedure of Hyperthermic Intraperitoneal Chemotherapy (HIPEC).

Around forty years ago, the first intracavitary chemotherapy strategies [8,9,10] were proposed for the clinical management of PC, relying on IP instillation of liquid antineoplastic drugs using an indwelling catheter after removing all macroscopic peritoneal metastases with cytoreductive surgery (CRS) [11,12,13]. Thus far, a variety of IP chemotherapy (IPC) techniques have been established, which are mostly classified according to the temperature or administration timing as follows: normothermic IPC, hyperthermic IPC (HIPEC), early postoperative IPC, and delayed postoperative IPC [14,15] (Figure 1C,D). The clinical benefit of combining surgical cytoreduction with fluid-based intraperitoneal chemotherapy in the management of PC has been well-evidenced for decades, although this combined treatment modality is associated with a considerable morbidity and a substantial proportion of patients have demonstrated a locally unresectable cancer [16,17]. Furthermore, it is postulated that the potential effectivity of IPC is restricted by well-documented pharmacological limitations, such as a poor penetration of the drug into peritoneal nodules and an inhomogeneous drug distribution throughout the abdominal cavity, resulting in an unsatisfying treatment outcome [18]. 

Recently, the nebulization of chemotherapeutics has proven to be a well-suited and efficacious drug delivery mode for the treatment of inoperable tumors and for the prevention of local tumor recurrence. Aerosolized chemotherapy has depicted utility in several cancers, particularly lung malignancies and peritoneal carcinomatosis [19,20,21,22,23]. Interestingly, nebulization presents attractive possibilities for localized cancer therapy through ensuring a homogenous drug distribution and optimal antitumor effects, in combination with lower required doses [20,21,22,24,25]. This review aims to shed light on the progress, challenges, and opportunities in employing cancer therapeutics for locoregional drug delivery, with a special emphasis on the nebulization approach for intraperitoneal therapies. Given the recent advances in aerosol chemotherapy as a locoregional drug delivery approach, we first present a new IP aerosolization strategy, referred to as Pressurized Intraperitoneal Aerosol Chemotherapy (PIPAC), where we discuss the potential pharmacological advantages of PIPAC along with the technical hurdles that need to be overcome to utilize this procedure in the clinical management of PC. Lastly, we focus on the nanotechnology-based IP therapeutic modalities and particularly highlight the challenges to the application of nanomedicine in IP nebulization therapy.

## 2. Aerosolized Drug Delivery of Chemotherapeutics

### 2.1. Principles and Proof of Concept of PIPAC

Over the past decades, aerosolization has established a foothold in cancer medicine, with a strong emphasis placed on lung cancer therapy. In 1993, one of the first clinical studies regarding the pulmonary administration of a chemotherapeutic (5-fluorouracil, 5-FU) was published, which documented the implementation of nebulization chemotherapy by the administration of anticancer agents through the inhalation of nebulized aerosols [22]. 

Lungs are regarded as appealing candidates for localized chemotherapy, in virtue of possessing a large surface area for the rapid deposition of therapeutics, extensive vascularization with a weak anatomical barrier, and a thin epithelial lining that does not limit the access to the possible lung cancer metastatic sites [26,27]. Inhalation chemotherapy in lung cancer, which allows for local delivery of the aerosolized chemotherapeutic agents to the site of disease, is non-invasive and could improve patient compliance [23,28,29]. However, it should be noted that the existing drug clearance mechanisms and bio-barriers in the respiratory airway systems, including mucus, ciliated cells, and resident macrophages, remain a challenge in the field of inhaled chemotherapy [30] and the development of innovative aerosolizable drug delivery technologies has been found to be an indispensable requirement in this field.

Besides locoregional delivery to the lungs, recent and exciting advances in nebulization chemotherapy have paved the way for the clinical application of a new, minimally-invasive intraperitoneal (IP) drug delivery technique, known as Pressurized Intraperitoneal Aerosol Chemotherapy (PIPAC). PIPAC benefits from the aerosolization approach for the treatment of patients diagnosed with end-stage cancers restricted to the peritoneal cavity.

The basic principles of the PIPAC procedure were developed and described in 2000 [31], while the first application in humans was introduced a decade later, in November 2011 [32,33,34,35]. The concept of PIPAC arose from the procedure performed during a diagnostic laparoscopy. Briefly, a constant 12-mm Hg capnoperitoneum is created through the insufflation of CO_2_ at 37 °C and two balloon trocars are introduced into the abdominal cavity (Figure 1A,B). A patented mono-component nozzle, the so-called PIPAC Micropump (MIP^®^, Reger Medizintechnik, Rottweil, Germany), is connected to a high-pressure injector with a high-pressure line and then inserted into the abdomen through one of the trocars. The liquid cytotoxic drug is nebulized with the MIP^®^ under a maximum pressure of 20 bars, generating a polydisperse aerosol in the abdominal cavity, with a mean droplet size of 25 µm. The system is maintained at a steady-state for 30 min at room temperature and the therapeutic capnoperitoneum is then evacuated via a closed aerosol waste system.

There is substantial in vitro, in vivo, ex vivo, and clinical evidence that PIPAC offers superior pharmacological advantages over the conventional intraperitoneal lavage [20,21,36] (Table 1). It has been reported that the chemo-aerosol, formed during the PIPAC procedure, is assumed to behave in a “gas-like” manner, and as a consequence, it could guarantee a homogenous distribution pattern of cytotoxic substances within the entire intraperitoneal space, which in turn enhances the area of peritoneal surface covered by the drug [20,21,37]. Furthermore, the artificial pressure gradient in the shape of a 12 mm Hg capnoperitoneum generated during PIPAC is assumed to counteract the elevated interstitial fluid pressure in tumor nodules, resulting in a superior drug penetration depth in peritoneal nodules in comparison with liquid IP chemotherapy [21,36]. Prior experimental studies have indeed confirmed that the increase in intraperitoneal pressure plays an important role in the absorption of fluid from the peritoneal cavity and seems to enhance the in-tissue drug influx upon intraperitoneal administration [38,39]. It has been shown by Solass and coworkers [20] that the penetration depth of chemotherapeutics into peritoneal nodules and tissue was 500–600 µm accompanied by high tissue drug concentrations (0.03–4.1 µmol/g), which is considerably higher than noted for peritoneal lavage with a liquid solution [39]. As such, PIPAC was able to introduce superior intratumoral drug concentrations with only 10% of the usual dose applied in HIPEC, leading to the regression of peritoneal nodules with limited hepatic and renal cytotoxicity. Furthermore, additional advantages are the possibility of performing PIPAC sequentially or in addition to traditional systemic chemotherapy, as the repeatability of the procedure can contribute to an enhanced treatment response and improved quality of life in patients [20,40,41].

While the results that have been obtained up to now are encouraging, there remain several obstacles to be overcome. Recently, published data collected from ex vivo and post-mortem animal investigations demonstrated a non-uniform spatial drug distribution pattern within the entire abdominal cavity during the PIPAC procedure. Granulometric characterization of the aerosol delivered by the MIP^®^ showed that 97.5 vol% of aerosol droplets are between 3–200 µm, which do not possess the suitable physical properties to provide a homogenous drug distribution [42,43,44]. Hence, such droplets are primarily deposited beneath the MIP^®^ nozzle on the peritoneum by gravitational settling and inertial impaction, creating extensive local aerosol “hot-spots”, rather than a homogenous distribution [42,45].

These findings are consistent with the observations obtained by other studies. Khosrawipour et al., for example, reported a remarkable variation in penetration depth between different regions of the abdominal cavity [44,46]. The highest uptake of the aerosolized cytotoxic agent into tumor nodules was observed in the opposite side and vicinity of the MIP^®^ nozzle with an in-tissue penetration depth of >311 µm compared to tissue samples located at more distant sites from the aerosol jet created by the MIP^®^. Therefore, the proximity of peritoneal tissue to the MIP^®^ could be regarded as a crucial parameter affecting the uptake of chemotherapeutics into peritoneal nodules during the PIPAC procedure [43,44,45].

Recently, new types of PIPAC methods have been developed to address the hurdles associated with this chemotherapy approach. For example, Jung Do et al. previously evaluated the feasibility and safety of combining the benefits of PIPAC technology with hyperthermia in a porcine model, known as hyperthermic PIPAC (H-PAC) [48]. Göhler et al. recently established an improved hyperthermic version of PIPAC in a swine model for the treatment of end-stage PC, termed hyperthermic intracavitary nanoaerosol therapy (HINAT) [47]. Here, the application of a nanoaerosol resulted in a more uniform drug distribution pattern and deeper in-tissue drug penetration in an intracavitary hyperthermic condition [47]. Finally, electrostatic PIPAC (ePIPAC) was developed, in which the electrostatic precipitation of the therapeutic aerosol was shown to improve the distribution pattern and tissue uptake of neoplastic agents in the peritoneal cavity, when compared to PIPAC alone [37,49]. Nevertheless, beyond technical innovations in the optimization of intra-abdominal aerosol therapy, PIPAC is still in its infancy and numerous challenges are expected. Furthermore, as PIPAC is only performed in patients who are no longer eligible for CRS and HIPEC, it should be considered as palliative care and CRS and HIPEC remain the standard of care for patients diagnosed in more early stages of the disease, despite their more invasive character (Figure 1C,D).

### 2.2. Clinical Studies on the Effectiveness of PIPAC

The PIPAC approach has offered new hope for the treatment of various peritoneal surface malignances (e.g., ovarian, gastric, and colorectal carcinomatosis), employing different anticancer substances such as cisplatin, oxaliplatin, and doxorubicin (Table 1). Given the success of PIPAC in preclinical studies, including in vivo [21] and ex vivo [36] models, Solass and co-workers attempted to investigate the first application of this treatment modality in human patients using cisplatin (7.5 mg/m^2^ body surface area) combined with doxorubicin (1.5 mg/m^2^). Despite an overall dose reduction, they documented significant antitumor activity by demonstrating high concentrations of doxorubicin in peritoneal nodules and tumor regression, even in platinum-resistant tumors with diminished renal and hepatic toxicity [20]. Another preliminary clinical study details the procedure and outcome of PIPAC with cisplatin and doxorubicin in 21 women with recurrent platinum-resistant ovarian cancer. In 3/21 of the selected patients, PIPAC was attempted; however, it could not be carried out owing to abdominal adhesions precluding laparoscopy required for this therapy. Therefore, further analyses were limited to 18 patients which underwent at least one PIPAC. Thirty-four PIPAC procedures were performed in these 18 patients in total, and in eight instances were combined with cytoreductive surgery. This investigation provided the clinical evidence that PIPAC was well-tolerated in most patients when applied without concomitant CRS and achieved an objective tumor response in 6/8 cases, with complete remission, partial remission, and stable disease observed in one, two, and three patients, respectively. A cumulative survival rate of 62% was noted 400 days after the beginning of treatment. This suggested that PIPAC may be considered as a palliative therapy option in women suffering from recurrent ovarian cancer, although further assessment in prospective clinical trials is needed [50].

Approximately one year later, the same group were included in a report on a phase 2 clinical study concerning the efficacy of PIPAC administered with cisplatin and doxorubicin in patients with recurrent, platinum-resistant ovarian, fallopian, or peritoneal cancer with peritoneal carcinomatosis. The authors concluded that PIPAC results in an objective tumor response, the induction of histological tumor regression coupled with an acceptable local and systemic toxicity, and the achievement of a clinical benefit rate of 62%. It was, however, envisaged that PIPAC still needs to be further examined in comparative clinical trials and other chemotherapy compounds as alternative combinations of cytotoxic agents may be valuable options for testing via the PIPAC approach [32].

Likewise, of note is a retrospective analysis, which was performed for the first time with the aim of exploring the efficacy of PIPAC with oxaliplatin (92 mg/m^2^) in recurrent, pretreated colorectal peritoneal metastasis. It was shown that PIPAC is capable of inducing an impressive response rate and objective tumor regression in 12 out of 14 patients (86%) in a salvage situation. The procedure was remarkably well-tolerated, with a limited local toxicity and indicated mean survival rate of 17.5 months after the first PIPAC application. It was suggested that the obtained results might provide the rationale for prospective, comparative clinical studies investigating the potential of PIPAC as a palliative therapy in patients with colorectal peritoneal metastasis who are not eligible for CRS and HIPEC [34]. Similarly, in an additional study by Nadiradze et al., PIPAC was conducted with low-dose cisplatin and doxorubicin, for the first time, in chemotherapy-resistant gastric peritoneal metastasis. The study documented an objective tumor response in half of the patients (12/24), including complete histological regression in six patients and a low incidence of severe adverse events. The obtained results were in accordance with previous observations reported in ovarian [32,50] and colorectal cancer [34] indicating encouraging survival data, although, as an exception, gastric cancer patients with malignant pleural effusion did not benefit from the treatment regimen. Therefore, it was proposed that the data should not be extrapolated to all patients with gastric peritoneal metastasis and additional research is needed in this area [33].

More recently, a case study by Graversen and co-workers focused on the therapy of pretreated peritoneal metastasis from a pancreatic origin through the application of PIPAC with low-dose cisplatin and doxorubicin. In this pilot study, the induction of histological regression and overall survival benefit suggested the activity and feasibility of PIPAC in these patients, although a larger study is required for validation [51]. In addition to previous investigations, it has recently been revealed by a retrospective cohort study that repetitive PIPAC procedures are feasible in the majority of patients with refractory carcinomatosis of various origins with a reduced incidence of intraoperative and postoperative complications, prompting the design of prospective analyses to monitor the oncological efficacy of this therapeutic strategy [57]. To systematically analyze the mechanism, pharmacokinetics, the safety and clinical efficacy of PIPAC, the available experimental and clinical evidence for this IP chemotherapy strategy with a special emphasis on ovarian cancer have been summarized in a recent review article [58]. In short, based on experimental studies, retrospective cohort studies, and clinical phase I and II trials reporting >3500 procedures in >1500 patients with PC of various primary malignancies, it is suggested that the management of PC with PIPAC is feasible and safe without systemic toxicity. Additionally, PIPAC maintains and/or improves the quality of life in patients with PC and recurrent cancers. Despite the clinical trial success in general, it is worth noting that further studies will help to elucidate the true potential of the PIPAC approach for the widespread implementation in a clinical setting. Furthermore, it is imperatively recommended that the efficacy of PIPAC in clinical practice should be compared to other treatment modalities (e.g. CRS, HIPEC, and systemic chemotherapy) in randomized phase III trials, as these studies are currently lacking.

## 3. Nanotherapeutics for Locoregional Cancer Therapy

### 3.1. Potential Benefits of Nanoparticulate Drug Delivery

The intrinsic limitations of conventional cancer therapeutics have ignited the growing interest in applying nanotherapies to cancer, substantially attributed to their appealing hallmarks for drug delivery, diagnosis, and imaging [59,60,61].

The fundamentals behind utilizing nanoscale delivery vehicles for cancer therapy stem from the fact that they afford extended circulation, reduced toxicity, controlled release, and enhanced drug protection compared to their free antineoplastic counterparts. Furthermore, it is widely believed that cancer nanotherapeutics can be further functionalized with targeting ligands to actively localize them at the tumor site, leading to improved therapeutic efficacy and reductions in systemic side effects by avoiding the delivery of cytotoxic drugs to nontumor cells [62]. Despite the enormous progress in the field of cancer nanomedicine, only a few nanotechnology-based therapeutic products, such as liposomes, albumin nanoparticles, and polymeric micelles, have been clinically approved by the Food and Drug Administration (FDA) for anticancer therapies so far, although more are in clinical trials [63]. Clearly, challenges lie ahead regarding chemistry, manufacturing, and commercialization before the clinical translation of a nanoparticle’s efficacy from preclinical animal models to humans can be made [64].

Over the last few decades, nanotechnology has also been considered for local drug delivery strategies. Notably, nanoparticles have become attractive candidates for IP drug delivery by virtue of their tunable physicochemical features (for example, their size, charge, shape and surface chemistries, and bioadhesive properties, among others), which facilitate bypassing the body clearance systems and the release of therapeutic payloads to the tumor site [19,65]. In addition, IP-administered nanovehicles not only have the ability to potentially lengthen the exposure of tumor nodules to chemotherapeutics, but also slow down the absorption to the systemic circulation, thus lowering systemic adverse effects [6,11,66,67,68]. Furthermore, nanoparticles (NPs) can be decorated with targeting ligands, which could largely assist in tumor accumulation, retention, and the cellular uptake of antineoplastic agents, while abrogating off-target effects [69,70,71,72,73]. The implementation of nanotherapeutics for localized cancer chemotherapy is largely guided by the desire to maximize the therapeutic concentrations and bioavailability of drugs nearby cancerous tissues over a longer period of time. We have previously reviewed the challenges in using nanomedicines for the treatment of peritoneal carcinomatosis [19], where nanoparticles are introduced in the peritoneal cavity through the injection of a solution or imbedded in controlled release hydrogel formulations [74,75,76]. In the following section, we will discuss the rationale behind using therapeutic nanocomplexes for intraperitoneal nebulization and provide an overview of existing challenges and future perspectives for the use of PIPAC for the local application of nanomedicine.

### 3.2. Challenges and Opportunities for the Nebulization of Nanomedicine in the Peritoneal Cavity

**Figure 2:**
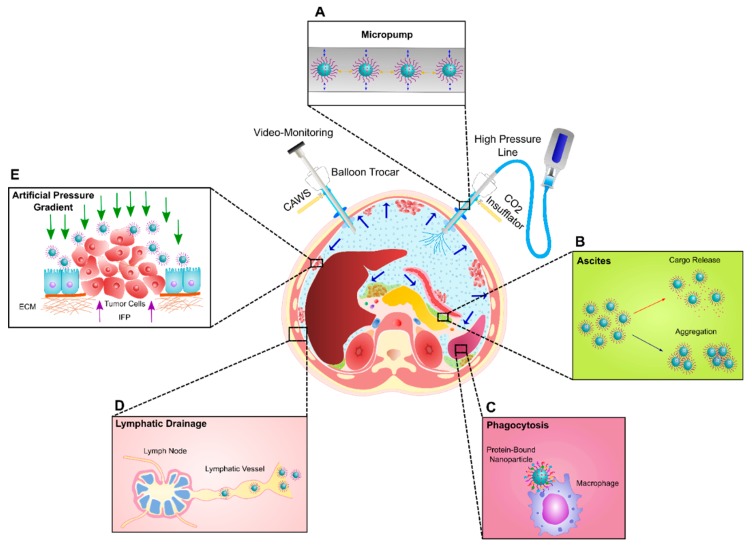
Schematic illustration of challenges to the intraperitoneal aerosolization of nanotherapeutics using Pressurized Intraperitoneal Aerosol Chemotherapy (PIPAC). (**A**) Colloidal stability of NPs against the pressures generated during the nebulization process. (**B**) Colloidal stability of NPs in the presence of biofluids (e.g., Ascites). (**C**) Adsorption of highly abundant proteins present in the peritoneal cavity on the surface of NPs and subsequent phagocytosis by resident macrophages in filtering organs (e.g., spleen and liver). (**D**) Lymphatic drainage of NPs. (**E**) Penetration of NPs into the peritoneal nodules through the creation of a high artificial pressure gradient counteracting the elevated interstitial fluid pressure (IFP) in tumors.

Provided that cancer nanomedicine in the IP drug delivery area continuously advances, a new opportunity may be created for exploiting the potential of nanoparticulate systems combined with the PIPAC procedure. It should be noted, however, that our current understanding of how nanoparticle systems behave when intraperitoneally nebulized through PIPAC is insufficient due to the very few research studies that have been devoted to exploring this subject. Herein, one of the first challenges is the colloidal stability of nanoparticle formulations when exposed to forces generated during IP nebulization (Figure 2A). Indeed, the ideal nanoscale systems should have the ability to be efficiently transferred into aerosols, but on the other hand, should endure a high aerosolization pressure to hinder any dramatic alterations in the structure, composition, and functionality. Problems which might occur under the influence of nebulization are nanoparticle aggregation, premature payload release, and the loss of targeting capability, which are all expected to result in limited therapeutic activity. Furthermore, in the case of hyperthermic PIPAC procedures, the chemical composition of NPs (in particular for heat-sensitive and lipid-based nanomaterials) should withstand the high aerosol temperature (41–43 °C) to prevent changes in nanoparticle formulations composed of low-melting-point materials.

Apart from the nanoparticle, the cargo it is carrying should also be able to withstand the high pressure during nebulization. For chemotherapeutics like doxorubicin, cisplatin, or paclitaxel, this is most likely less of an issue, given their low molecular weight and simple chemical structure. Macromolecules like proteins, antibodies, and nucleic acids, however, might be more vulnerable to structural alterations during nebulization as subtle changes in the 3D structure and folding of these molecules can already have a consequence on their biological activity. In a recent in vitro study, however, Minnaert et al. demonstrated that nebulization is an appealing approach that can be used to distribute Lipofectamin RNAiMAX/siRNA complexes in the peritoneal cavity, which maintain their transfection efficiency upon aerosolization [52]. Furthermore, we have recently demonstrated that high-pressure nebulization of messenger Max/mRNA complexes to the peritoneal cavity of adult athymic nude rats is feasible, leading to a homogenous distribution of luciferase mRNA expression in the peritoneal cavity. Additionally, when compared to an IP injection, the aerosolization approach seemed to result in a more uniform mRNA expression which covered a larger surface area of the peritoneum [53]. Therefore, these first in vivo results demonstrate that nucleic acids seemed to survive the high pressure that was applied during the nebulization procedure. Nevertheless, further in vivo validation is indispensable to provide more insights into the challenges faced during the combined application of nanotechnology-based drug delivery systems and the IP aerosolization strategy.

Another formulation that might be useful for IP nebulization is Abraxane, an FDA-approved nanoparticle formulation of paclitaxel (PTX), consisting of 4–14 nm PTX-albumin aggregates that form an albumin-PTX nanoparticle of roughly 130 nm in diameter [77]. The strengths of Abraxane have been demonstrated through the activity in metastatic breast, pancreatic, and non-small cell lung cancers. It is also noteworthy that recent preclinical and clinical studies have focused on the IP administration of Abraxane for the treatment of platinum-resistant PC due to the superior antitumor activity, favorable toxicity profile, and better overall response and survival compared with conventional IV administration [78,79,80,81]. In this regard, pressurized IP aerosol administration of Abraxane has been proposed by a recent phase I clinical study in patients with unresectable peritoneal metastasis from upper gastrointestinal, ovarian, or breast malignancies, aiming to determine the maximum tolerated dose (MTD) of Abraxane for prospective randomized phase II clinical trials of PIPAC therapy [54]. To the best of our knowledge, this is the first in vivo study in humans trying to elucidate the feasibility and added value of using PIPAC for the administration of a nanoparticle formulation, yet the outcome still has to be determined. Apart from Abraxane, no other FDA-approved nanomedicines have been evaluated for the treatment of peritoneal metastasis so far. There are about 50 FDA-approved nanomedicines which are categorized as liposomal formulations, polymeric formulations, nanocrystals, or drug conjugates [64,65,82,83]. Only a few of them are useful for the treatment of cancer (e.g., Doxil), but none of them have been specifically approved for intraperitoneal application. There are nanomedicines currently being evaluated in preclinical and clinical studies for IP cancer therapies (e.g., Nanotax and Nano-platin), which could have future potential for IP application through PIPAC (Table 1). The use of a liposomal nanoformulation of cisplatin (e.g., Lipoplatin), however, is less likely to be successful as encapsulated cisplatin showed a decreased efficacy when compared to the free drug [84].

Nevertheless, it should be noted that the particular challenges that occur upon the IP injection of nanomedicines will most likely remain after the nebulization of NPs in the peritoneal cavity. Indeed, on the first contact of nebulized nanotherapeutics with the biological environment of the peritoneal cavity, deposited particles are exposed to several potential bio-barriers. Importantly, aerosolized NPs can come into contact with ascites fluid, which accumulates in the peritoneal cavity at an advanced stage of PC. Considering the formation of even a limited amount of ascites fluid in patients with peritoneal metastasis, who are eligible for the PIPAC procedure, it will be crucial to identify the therapeutic efficacy of nebulized nanotechnology-based drug delivery carriers in ascites fluid in the relevant in vitro settings before in vivo testing is pursued. In this regard, we have established an advanced microscopy technique which has afforded us the unique possibility to explore the colloidal stability of nanotechnology-based platforms in undiluted biofluids (e.g., ascites, blood, serum, and plasma), which are also likely to be applicable for tracing the biological behavior of NPs following in vivo administration [85]. By means of the fluorescent single particle tracking (f-SPT) method, we are able to determine whether or not NPs remain colloidally stable (e.g., do not aggregate) and if the premature release of cargo does not occur (Figure 2B). Indeed, biofluids such as ascites fluid have a high protein content of which the negatively charged albumin might compete with the binding of negatively charged cargo such as nucleic acids to the nanoparticles. Using SPT, we recently reported that liposome-siRNA nanocomplexes were colloidally stable in the presence of ascites fluid obtained from patients with PC, although their cellular uptake and ability to silence the target genes in the SKOV3 cell line were substantially diminished [86].

Furthermore, when nanomedicines encounter biological fluids, different biomolecules (typically proteins) present in these biofluids can be adsorbed by the nanoparticles, thereby immediately covering their surface. This gives rise to the formation of a “protein corona”, which can alter the physicochemical characteristics of NPs (for example, size, stability, and surface properties) and, more importantly, dictates the biological responses that nanoparticles elicit, such as their cellular uptake, biodistribution, and toxicity [87,88,89,90,91,92]. Additionally, it has become increasingly clear that the presence of this protein corona can trigger recognition and phagocytosis of the nanoparticles by resident macrophages of the mononuclear phagocyte system (MPS), located in filtering organs (i.e., the liver and spleen) (Figure 2C). There exists empirical evidence suggesting that the size of IP-administered delivery vehicles can largely control their peritoneal retention, in view of the fact that the majority of nano-sized drug delivery systems are rapidly cleared from the peritoneum and trafficked to the spleen and liver, where they are exposed to macrophage clearance. Conversely, microparticles have revealed a prolonged residence time in the peritoneal cavity, although the high incidence of peritoneal adhesions induced by microparticles over a longer period of time may rule out their therapeutic application for long-term IP therapy [93].

Besides macrophage clearance, a growing emphasis has been placed on the role of the lymphatic system as a major factor in peritoneal clearance, which is to a great extent related to the micron-sized diameter of lymphatic duct openings (in a murine model), hence particle size criteria come into play once again (Figure 2D). It has been documented that small nano-sized formulations (less than 500 nm) are readily drained through the lymphatic ducts and easily pass the lymph nodes to reach the system circulation, whereas in the case of larger delivery vehicles, if their size approaches the size of lymphatic duct openings, drainage via the lymphatic system is limited and they are mostly entrapped in the lymph nodes instead [81,94,95,96,97].

In addition to particle size, the shape of therapeutic nanoparticles may be an important determinant of IP therapy. Compared to their spherical counterparts, elongated nanostructures (for instance, nanoworms and nanorods) with adequate geometric dimensions are thought to hamper the macrophage clearance, as well as lymphatic drainage, thereby extending the residence time in the peritoneal cavity [98,99,100,101,102]. It is also assumed that the high aspect ratio of non-spherical carriers may allow more efficient deposition and superior coverage of the peritoneal surface following IP nebulization. Likewise, the surface modification (e.g., Polyethylene Glycol (PEG) grafting) and charge (i.e., positive charge) of a delivery vehicle also seem to be key to enhancing the peritoneal retention, by circumventing phagocytosis by macrophages [103,104,105].

Finally, the penetration of nebulized nanotherapeutics to the peritoneal nodules poses an additional challenge to IP drug delivery. The tumor microenvironment is characterized by a dense extracellular matrix composed of collagen fibers and several different proteins [3,13]. Furthermore, the elevated interstitial fluid pressure (IFP) caused by vascular hyperpermeability and the lack of functional lymphatic vessels inside the tumor tissue, contribute to the poor penetration of NPs into the tumor [3,13,106,107,108]. It is widely supposed that the high intra-abdominal pressure created during PIPAC counteracts the interstitial hypertension in tumor nodules, providing a deeper in-tissue drug penetration and superior antitumor efficacy for chemotherapeutics (Figure 2E). However, whether or not PIPAC can also enhance the tumor penetration of nanoparticles remains unclear. One can imagine, however, that the acceleration of nanoparticles during the nebulization procedure might help to ‘shoot’ them deeper into tissue layers. Therefore, exploring the impact of the NP physicochemical properties (e.g., size, charge, surface features, and geometry) on tumor penetration [101,102,109,110,111,112,113,114], will be crucial to exploiting the potential of nanomedicine administration with the PIPAC approach. Interestingly, the balloon trocars used in the PIPAC procedure allow the application of laser light into the peritoneal cavity. This opens up the avenue for low-dose photodynamic therapy (PDT), which has been proven to result in a selective uptake increase of anticancer drugs in tumor nodules in the thoracic cavity [115]. Whether or not PIPAC offers benefits to administer more advanced formulations for controlled IP drug delivery (e.g., in-situ cross-linkable hydrogels containing NPs loaded with chemotherapeutic agents, Near Infrared (NIR)-triggered drug release from NPs for controlled IP drug delivery, or low-dose PDT [65,116,117,118]) remains to be seen.

## 4. Conclusion and Future Directions

Over the past years, the utility of locoregional chemotherapy has attracted the attention of clinicians to open the door to new approaches for the clinical management of PC, enabling them to address some of the shortcomings typically associated with systemic palliative chemotherapy. Notably, local drug delivery strategies could enhance the therapeutic responses to tumor eradication, by facilitating the direct delivery of anti-neoplastic agents to the tumor site, thus mitigating the systemic adverse effects.

Given the great promise shown by aerosolized drug delivery strategies for localized cancer therapy, an innovative intra-abdominal aerosol chemotherapeutic approach, known as PIPAC, was recently introduced to the IP therapy regimens. By benefiting from the physical properties of gas and pressure, PIPAC may present new opportunities to the area of IP drug delivery compared to conventional liquid IP chemotherapy, resulting in a more homogenous drug distribution pattern, together with deeper drug penetration into tumor nodules within the peritoneal space. However, an issue that remains clear is that PIPAC is still in its infancy and must mature enough to fulfill its potential in improving the patient survival.

Furthermore, with the emergence of nanotechnology in the past few decades, a new wave of utilizing nanomaterials has begun in IP cancer therapy, opening up several new possibilities in this field ranging from an extended retention time and sustained drug release in the peritoneal cavity to targeted drug delivery to peritoneal tumor nodules, among others. Although some of these opportunities afforded by nanotechnology to the IP therapies may become realities in the foreseeable future, there is still ample room to improve the development of clinically efficacious nanosystems for the treatment of PC. The key to designing the next generation of nanotherapeutics for aerosol IP therapies heavily involves a much deeper understanding of how nebulized nanoscale vehicles interact with the complex biological environment of the peritoneal cavity and invoke biological responses which can in turn determine the fate of nanoparticle-based drug delivery systems in vivo. Barriers to overcome include drainage through the lymphatic system, phagocytosis by macrophages, and tumor accumulation and penetration. Additionally, the combination with more advanced formulations for controlled drug delivery could result in a more successful translation of aerosolized nanotherapeutics from a benchtop to bedside, ensuring an enhanced quality of life for patients with PC. Nevertheless, we should keep in mind that local drug delivery may not always be sufficient as a stand-alone approach, but can be applied in combination with existing and emerging therapeutic strategies to offer a potential benefit for enhancing the patient outcomes.

## Figures and Tables

**Table 1:** Examples of preclinical and clinical studies on the efficacy of Pressurized Intraperitoneal Aerosol Chemotherapy (PIPAC).

Active Pharmaceutical Compounds	Type of PIPAC	Experimental Model/Ex Vivo, In Vivo, Clinical Trial	Cancer Type	Outcome	Reference
**1. Tracers and chemotherapeutics**					
Doxorubicin	PIPAC	Pig/ex vivo	Fresh tissue samples of swine peritoneum	The study evidenced a heterogeneous drug distribution pattern of ex vivo PIPAC, indicating a significantly higher penetration depth of the drug in tissues directly exposed to the aerosol jet in comparison with the ones placed on the side wall and the top of the box.	[43]
Doxorubicin	PIPAC	Pig/ex vivo	Fresh postmortem swine peritoneum	Ex vivo data suggested that a higher drug dose and a closer positioning of the Micropump towards the tissue samples could assist in a superior drug penetration. Nevertheless, an increase in internal pressure did not significantly change the penetration depth of doxorubicin. Furthermore, changing the drug concentration and position of the nozzle, as well as increasing the pressure, cannot noticeably give rise to a more homogenous drug distribution pattern.	[46]
Methylene blue	PIPAC	Pig/in vivo	Healthy animal	Significant improvement in both the biodistribution and penetration of the test substance in the peritoneal cavity in a large animal model compared to peritoneal lavage.	[21]
Methylene blue	PIPAC	Pig/in vivo	Healthy animal	First description and development of basic principles of the PIPAC procedure in vivo. This study showed that all exposed peritoneal surfaces were stained by the methylene blue, indicating the partial distribution of this active substance within the peritoneal cavity.	[31]
^99m^Tc-Pertechnetate (isotopes of the radioactive element technetium)	PIPAC	Pig/in vivo	Healthy animal	Using scintigraphic peritoneography, this study revealed the inhomogenous intra-abdominal distribution pattern of aerosol during the PIPAC procedure in a postmortem swine model.	[45]
Tracer substances (toluidine blue and DT01)	ePIPAC	Pig/in vivo	Healthy animal	Obtained data showed that electrostatic precipitation of aerosolized substances was technically feasible in all electrostatic PIPAC (ePIPAC) animals. Generally, ePIPAC affords homogenous staining of peritoneal surfaces and enhanced tissue uptake of tracers (up to tenfold) compared to PIPAC.	[37]
^99m^Tc-Pertechnetate/Doxorubicin/Methylene blue	HINAT	Pig/in vivo	Healthy animal	Hyperthermic intracavitary nanoaerosol therapy (HINAT) was introduced as an enhanced approach of PIPAC, generating hyperthermic unipolar-charged nanosized aerosols which can in turn provide a more uniform drug deposition throughout the peritoneal cavity along with significantly deeper drug penetration.	[47]
Indocyanine green/Cisplatin	H-PAC	Pig/in vivo	Healthy animal	In this study, a constant hyperthermic capnoperitoneum was created using a heating apparatus. The feasibility and safety of hyperthermic PIPAC (H-PAC) were shown in the porcine survival model.	[48]
Doxorubicin	PIPAC	Pig/in vivo	Healthy animal	Aerosolized drugs can reach all areas within the peritoneal cavity, although the highest penetration depth was observed in the peritoneal surfaces around the Micropump.	[44]
Doxorubicin + Cisplatin	PIPAC	Human/clinical trial	Advanced PC from gastric, appendiceal, and ovarian origin	Superior antitumor activity with a high local concentration and low systemic toxicity. Regression of peritoneal carcinomatosis (PC) in chemo-resistant tumors was observed using 10% of an usual systemic dose.	[20]
Doxorubicin + Cisplatin	PIPAC	Human/clinical trial	Recurrent, platinum-resistant ovarian, fallopian, or peritoneal cancer with peritoneal carcinomatosis	Safety and activity of PIPAC were assessed in patients. This approach resulted in an objective tumor response and histological tumor regression along with acceptable local and systemic toxicity. In general, 62% of patients in this trial achieved the clinical benefit.	[32]
Doxorubicin + Cisplatin	PIPAC	Human/clinical trial	Gastric peritoneal metastasis	PIPAC procedure was safe and induced an objective tumor response in 50% of selected patients.	[33]
Oxaliplatin	PIPAC	Human/clinical trial	Colorectal peritoneal metastasis	This retrospective analysis revealed that repeated PIPAC can induce considerable pathological responses in pretreated colorectal peritoneal metastases while causing less local toxicity.	[34]
Oxaliplatin/Doxorubicin + Cisplatin	PIPAC	Human/clinical trial	Peritoneal carcinomatosis	Safety and feasibility of PIPAC associated with systemic chemotherapy were explored. Preliminary data showed that this combination therapy did not lead to significant renal and hepatic toxicity. This combined treatment may be beneficial for patients with extraperitoneal disease or those at a high risk of disease development.	[35]
Doxorubicin + Cisplatin	PIPAC	Human/clinical trial	Peritoneal carcinomatosis	A retrospective cohort study assessed the objective tumor response in a pretreated population of women with peritoneal carcinomatosis. Results showed that PIPAC can preserve the quality of life; however, appropriate patient selection in terms of performance status and the number of previous surgeries should be take into consideration.	[41]
Doxorubicin + Cisplatin	ePIPAC	Human/clinical trial	Peritoneal metastasis of hepatobiliary-pancreatic origin	These preliminary results in human patients suggested that ePIPAC is well-tolerated and technically feasible and can induce the regression of biologically aggressive tumors.	[49]
Doxorubicin + Cisplatin	PIPAC	Human/clinical trial	Recurrent, platinum-resistant ovarian cancer	A preliminary clinical report on the activity of PIPAC provided evidence that PIPAC is well-tolerated in most patients and achieved an objective tumor response in 6/8 cases.	[50]
Doxorubicin + Cisplatin	PIPAC	Human/clinical trial	Peritoneal metastasis from pancreatic cancer	This case study reports on the activity of PIPAC through the induction of histologic regression in pretreated patients with systemic chemotherapy.	[51]
**2. Biomolecules and Nanoparticles**					
Dbait (noncoding DNA fragments) coupled to cholesterol molecule and Cy5	PIPAC	Human/ex vivo	Human sample of peritoneal carcinomatosis from an endometrial adenocarcinoma	The fluorescence signal was detected in the tumor nodules up to 1 mm in depth following aerosolization. On the contrary, no uptake was observed in the lavage sample.	[36]
Lipofectamin RNAiMAX/siRNA complexes	PIPAC	Human/ in vitro	Luciferase expressing ovarian cancer cell line (Luc SKOV3)	This study revealed the pronounced in vitro stability of complexes in ascites, as well as the noticeable transfection efficiency upon nebulization.	[52]
messengerMax/ Luc mRNA complexes	PIPAC	Rat/ in vivo	Healthy animal	First study demonstrating the feasibility of high-pressure nebulization of mRNA complexes to the peritoneal cavity of rats, affording a more homogenous distribution of luciferase mRNA expression in the entire peritoneal cavity compared to intraperitoneal injection.	[53]
Abraxane (albumin-bound paclitaxel) (FDA approved)	PIPAC	Human/clinical trial	Unresectable peritoneal metastasis from upper gastrointestinal, ovarian, or breast malignancies	Outcome to be determined.	[54]
**3. Nanoformulations potentially suitable for future implementation in PIPAC**					
Nanotax^®^ (nanoparticle formulation of paclitaxel)	IP administration	Human/clinical trial	Peritoneal malignacies	Compared to IV-administered paclitaxel, intraperitoneal (IP) administration of Nanotax® gives rise to higher paclitaxel (PTX) concentrations in the peritoneal cavity for a prolonged period of time, along with minimal sytemic exposure and a reduced toxicity.	[55]
1. Nano-taxol (liposomal Paclitaxel) 2. Nano-platin (polymeric micelle cisplatin) 3. Nano-topotecan (polymeric micelle topotecan) 4. Doxil (pegylated liposomal Doxorubicin) (FDA approved)	IP administration	Mouse/in vivo	Peritoneal metastasis of human ovarian cancer cell line (ES-2-luc)	This study indicated that systemic delivery of all tested nanomedicines failed to present a superior therapeutic efficacy compared with each free drug counterpart. In addition, IP delivery of these nanotherapeutics demonstrated a better antitumor activity only for Nano-taxol and Nano-topotecan when compared to corresponding free drugs. It is assumed that the structure of Abraxane and Doxil is considerably stable to release the encapsulated drug in the peritoneal cavity for the effective treatment of PC.	[56]
